# An improved pre-clinical patient-derived liquid xenograft mouse model for acute myeloid leukemia

**DOI:** 10.1186/s13045-017-0532-x

**Published:** 2017-10-06

**Authors:** Zhisheng Her, Kylie Su Mei Yong, Kathirvel Paramasivam, Wilson Wei Sheng Tan, Xue Ying Chan, Sue Yee Tan, Min Liu, Yong Fan, Yeh Ching Linn, Kam Man Hui, Uttam Surana, Qingfeng Chen

**Affiliations:** 1 Institute of Molecular and Cell Biology, Agency for Science, Technology and Research (A*STAR), Proteos, 61 Biopolis Drive, Singapore, 138673 Singapore; 2 Department of Medicine, Yong Loo Lin School of Medicine, National University of Singapore, Singapore, Singapore; 3 Key Laboratory for Major Obstetric Diseases of Guangdong Province, The Third Affiliated Hospital of Guangzhou Medical University, Guangzhou, 510150 China; 4 Department of Haematology, Singapore General Hospital, Singapore, Singapore; 5 Division of Cellular and Molecular Research, National Cancer Centre, Singapore, Singapore; 6 Department of Pharmacology, National University of Singapore, Singapore, Singapore; 7 Bioprocessing Technology Institute, Agency for Science, Technology and Research, Singapore, Singapore; 8 Department of Microbiology and Immunology, Yong Loo Lin School of Medicine, National University of Singapore, Singapore, Singapore

**Keywords:** Acute myeloid leukemia, Patient-derived xenograft, Leukemic stem cells, Tyrosine kinase inhibitors

## Abstract

**Background:**

Xenotransplantation of patient-derived AML (acute myeloid leukemia) cells in NOD*-scid Il2rγ* ^*null*^ (NSG) mice is the method of choice for evaluating this human hematologic malignancy. However, existing models constructed using intravenous injection in adult or newborn NSG mice have inferior engraftment efficiency, poor peripheral blood engraftment, or are difficult to construct.

**Methods:**

Here, we describe an improved AML xenograft model where primary human AML cells were injected into NSG newborn pups intrahepatically.

**Results:**

Introduction of primary cells from AML patients resulted in high levels of engraftment in peripheral blood, spleen, and bone marrow (BM) of recipient mice. The phenotype of engrafted AML cells remained unaltered during serial transplantation. The mice developed features that are consistent with human AML including spleen enlargement and infiltration of AML cells into multiple organs. Importantly, we demonstrated that although leukemic stem cell activity is enriched and mediated by CD34^+^CD117^+^ subpopulation, CD34^+^CD117^−^ subpopulation can acquire CD34^+^CD117^+^ phenotype through de-differentiation. Lastly, we evaluated the therapeutic potential of Sorafenib and Regorafenib in this AML model and found that periphery and spleen AML cells are sensitive to these treatments, whereas BM provides a protective environment to AML.

**Conclusions:**

Collectively, our improved model is robust, easy-to-construct, and reliable for pre-clinical AML studies.

**Electronic supplementary material:**

The online version of this article (10.1186/s13045-017-0532-x) contains supplementary material, which is available to authorized users.

## Background

Acute myeloid leukemia (AML) is one of the most common types of leukemia and accounts for ~ 42% of all leukemic deaths [1]. This has warranted a huge focus on its pathogenesis and disease management, as compared to other types of leukemia. Acute myeloid leukemia, marked by abnormal proliferation and differentiation of myeloid leukemic stem cells, is genetically and biologically heterogeneous [2]. Uncontrolled proliferation of leukemic stem cells forms leukemic blasts in the BM and peripheral blood circulation that eventually results in BM failure and deaths [2]. Despite a better understanding of the genetic aberrations [3, 4] that contribute to AML and recent therapeutic advances [5], the overall five-year survival rate remains low at 30–40% in patients younger than 60 years and less than 20% for patients above 60 years [6].

Therefore, there is a need to develop relevant AML animal models for the purpose of novel targets discovery and assessment of new therapies. Since the early 1900s, murine models have been extensively used to study AML, using approaches such as carcinogen-induced transplantable models, transgenic, xenograft, and mosaic models [7]. In particular, xenograft of patient-derived AML cells into immunodeficient mice such as severe combined immunodeficient (SCID) [8], non-obese diabetic (NOD)/SCID [9], and NOD-*scid Il2rγ* ^*null*^ (NSG) [2] mice was instrumental in defining leukemic stem cells [8] and their chemotherapy-resistant properties [2, 10]. Due to their longer life span (> 90 weeks) and greater engraftment capacity, NSG mice are the most widely used animal model [9, 11, 12].

While xenograft AML model can provide novel insights in understanding human AML biology, a vast improvement in existing models is desired. Often, construction of xenograft models relies on technically challenging methods such as neonatal craniofacial intravenous injection in neonatal mice [2] and intratibial or intrafemoral injections in adult mice [13–15]. In addition, the use of adult mice resulted in significantly lower engraftment capacity compared to newborn pups, hence, hindering long-term evaluation [2]. Importantly, existing AML models that utilize adult mice exhibit limited peripheral blood engraftment [11], a hallmark feature of human AML. Therefore, there is a need for an AML xenograft model that is easier to construct, adequately recapitulates human AML, and allows for long-term evaluation in vivo.

In this study, we sought to establish an improved pre-clinical AML xenograft model that is robust and easier to construct as compared to existing models. Using BM mononuclear cells obtained from seven AML patients, T cell-depleted AML cells were injected into sublethal irradiated NSG newborn pups via the intrahepatic route, a method routinely used in the humanization of NSG mice [16]. Three (Leu 14, BMI 1690, and BMI 1808) out of the seven AML patients exhibited AML leukemic blasts-associated phenotype and successfully engrafted in NSG recipient mice. Cytometric and histological analysis revealed high level of AML engraftment in the peripheral blood, spleen, and BM of recipient NSG mice. Serial transplantation, up to tertiary transplantation, was performed to further characterize our model. We demonstrated that CD34^+^ cells have significantly greater engraftment capacity than CD34^−^ cells. Furthermore, CD117 expression on CD34^+^ cells enhanced engraftment level. When compared to the existing model constructed using NSG adult mice and intravenous injection, our method showed more efficient AML engraftment. Lastly, the therapeutic potential of multi-kinase inhibitors Sorafenib and Regorafenib against AML was evaluated in our model. The favorable outcome of Sorafenib and Regorafenib was recapitulated in our model, with AML cells in the periphery and spleen sensitive to treatments, while those in BM remained unaffected. Collectively, our model serves as a robust, easy-to-construct and reliable pre-clinical tool for AML that will facilitate the discovery of new targets and assessment of new therapeutics.

## Methods

### Cell preparation

Bone marrow cells were obtained from patients with acute leukemia who had marrow study done at the time of diagnosis. Patients gave informed consent for additional aliquot of the marrow aspirate to be used for research purposes in accordance with the ethical guidelines of Singapore General Hospital. Patients with AML were diagnosed using the French-American-British (FAB) classification system; subtype M1 (patients Leu 32 and BMI 1786), M4 (patient BMI 1808), M5 (patient BMI 1690), and M5a (patients Leu 14, Leu 29, and Leu 33). Bone marrow cells were processed using ficoll density gradient centrifugation to isolate mononuclear cells. Cells were frozen and stored in liquid nitrogen until use.

### Mice

NOD-*scid Il2rγ* ^*null*^ (NSG) mice were purchased from The Jackson Laboratory. All mice were bred and kept under specific pathogen-free conditions in Biological Resource Centre, Agency for Science, Technology and Research, Singapore. All experiments and procedures were approved by the Institutional Animal Care and Use Committee (IACUC) of the Agency for Science, Technology and Research, Singapore, in accordance with the guidelines of the Agri-Food and Veterinary Authority and the National Advisory Committee for Laboratory Animal Research of Singapore.

### Primary and serial xenotransplantation of AML cells

For primary xenotransplantation, BM mononuclear cells were depleted of CD3^+^ cells using PE selection kit (STEMCELL Technologies) upon labeling with PE-conjugated mouse anti-human CD3 antibody (Biolegend) according to manufacturer’s instructions. One to 3-day-old NSG pups were sub-lethally irradiated at 1Gy and engrafted with 8.7 × 10^4^–7.9 × 10^5^ of CD3-depleted AML mononuclear cells from seven AML patients (Leu 14, Leu 29, Leu 32, Leu 33, BMI 1690, BMI 1786, and BMI 1808) via intrahepatic injection route. As described previously [17], intrahepatic inoculation was performed by maintaining the irradiated NSG pup in posterior position (face-up) between the thumb and index finger to expose the abdomen and the liver, which is visible on the right flank. An insulin syringe loaded with 50 μl of cell mixture was then held perpendicular to the pup body by the other hand and inserted straight into the pup liver with bezel facing upwards to release the contents. Mice were bled submandibularly to evaluate the engraftment of AML cells in peripheral blood at 2–4-week intervals after week 6 post-engraftment using flow cytometry. Engraftment of AML cells in BM and spleen was evaluated at endpoint (week 12–20 post-engraftment) using flow cytometry. Cells from BM and spleen were pooled and used for serial xenotransplantation.

Secondary and tertiary xenotransplantation were evaluated on pooled BM and spleen cells from Leu 14. For serial xenotransplantation, CD34^+^ cells from pooled BM and spleen cells were purified with either fluorescence-activated cell sorting (FACS) using FACSAria (BD Biosciences) after labeling with fluorochrome-conjugated mouse anti-human CD45 (Biolegend) and anti-human CD34 (BD Biosciences) monoclonal antibodies or by magnetic-sorting using CD34 positive selection kit (STEMCELL Technologies) according to manufacturer’s instructions. The purity of human CD34^+^ cells was > 95% after FACs or magnetic-sorting. Cell number range from 1 × 10^4^–5 × 10^5^ cells were injected into irradiated NSG recipients. Xenotransplantation in NSG adult mice (6-week-old) was performed via tail-vein intravenous injection after sublethal irradiation at 2.5Gy.

### Immune cell isolation from peripheral blood, spleen, and BM

Peripheral blood was collected submandibularly from mice in EDTA tube (Greiner Bio-One). Red blood cells (RBCs) were lyzed using RBC lysis buffer (Life Technologies) prior to flow cytometry analysis. For spleen and BM, tissues were meshed and cell contents from femur and tibia were flushed using a syringe, respectively. Cell debris was removed by passing contents through 70 μm cell strainer (Thermo Fisher Scientific). RBCs were further lyzed and contents passed through 70 μm cell strainer prior to flow cytometry analysis and storage.

### Flow cytometry analysis of peripheral blood, spleen, and BM

Live immune cells from peripheral blood, spleen, and BM were determined by staining with live/dead fixable blue dead cell stain kit (Life Technologies) for 30 min prior to cell-specific marker labeling. Cells were labeled with anti-human CD34 (clone 581; BD Biosciences), anti-human CD3 (UCHT1; Biolegend), anti-human CD56 (MEM-188, Biolegend), anti-human CD14 (63D3; Biolegend), anti-human CD19 (SJ25C1; BD Biosciences), anti-human CD117 (104D2; Biolegend), anti-human CD38 (HB-7; Biolegend), anti-human CD33 (WM53; BD Biosciences), mouse CD45.1 (A20; BD Biosciences), anti-human CD8 (SK1; Biolegend), anti-human CD4 (SK3; BD Biosciences), and anti-human CD45 (HI30; Biolegend) monoclonal antibodies for 30 min at room temperature. After incubation, cells were washed and resuspended in FACs buffer containing phosphate buffered saline (PBS), 0.2% bovine serum albumin (GE Healthcare Life Sciences), and 0.05% sodium azide (Merck) for flow cytometry data acquisition. Data was acquired using a LSR II flow cytometer (BD Biosciences) with FACSDiva software, and analysis was performed using FlowJo software (version 10; Tree Star Inc). Absolute count of cells in peripheral blood was determined using CountBright™ Absolute Counting Beads (Thermo Fisher Scientific).

### Hematoxylin & Eosin stain and immunohistochemistry

Multiple organs including brain, heart, lungs, liver, kidneys, forelimbs/hind limbs, and spleen were removed from sacrificed mice at endpoint. The organs were fixed in 10% formalin, embedded in paraffin wax, processed to obtain 5 μm sections, and subjected to Hematoxylin & Eosin (H&E) (Thermo Fisher Scientific) or immunohistochemistry staining following established protocols. Primary antibodies including anti-human CD45 (cat# ab781), anti-human MPO (cat# ab134132), and anti-human c-kit (cat# ab32363) monoclonal antibodies were purchased from Abcam and used for immunohistochemistry. The primary antibody was detected using Rabbit specific IHC polymer detection kit HRP/DAB (AbCam) or Mouse on Mouse Polymer IHC Kit (AbCam) following manufacturer’s instructions. Histopathological images were acquired using Axio Scan. Z1 slide scanner (Zeiss) and analyzed using Zen 2 (blue edition; Zeiss) software.

### Regorafenib and Sorafenib treatment

Sorafenib tosylate (Nexavar®; Bayer Healthcare Pharmaceuticals Inc) and Regorafenib (Stivarga®; Bayer HealthCare Pharmaceuticals Inc.) tablets were crushed and dissolved in isotonic saline water (B. Braun Medical Inc). Successfully engrafted mice with more than 30 human CD45 cells per microliter of blood (between week 12 and 16 post-engraftment) were randomly assigned to either untreated Regorafenib or Sorafenib treatment groups. Mice were given a daily dose of Regorafenib (5 mg/kg body weight) or Sorafenib (10 mg/kg body weight) via oral gavage and monitored for 1 month.

### Statistical analysis

Statistical analysis was performed using GraphPad Prism 5.0 software (GraphPad Software Inc). Pairwise comparison was performed using two-tailed Mann Whitney *U* test. *P* value less than 0.05 is considered statistically significant. All data are represented as mean ± standard error of mean (SEM).

## Results

### Immunophenotypic analysis of AML in BM mononuclear cells from seven AML patients

It is well documented that flow cytometric analysis using CD45/SSC gating can distinguish leukemic blast cells (low CD45 expression) from normal hematopoietic cell types (high CD45 expression) [18]. As shown by previous publications, CD33 (myeloid cell marker), CD34 (primitive stem cell marker), CD117 (c-kit receptor), and CD38 (cell activation marker) are key AML leukemic blasts-associated markers [19–21]. To confirm the presence of AML leukemic blasts in seven individuals with AML, BM mononuclear cells were isolated, labeled with anti-human CD34, CD117, CD38, CD33, and CD45 monoclonal antibodies, and analyzed using flow cytometry (Additional file 1: Figure S1a and Table 1). Out of the seven individuals with AML, samples from five individuals contained more than 80% leukemic blasts based on CD45^lo^ expression (Table 1). Although all samples expressed CD33, only three samples (Leu 14, BMI 1690, and BMI 1808) expressed CD33 within the CD45^lo^ compartment, suggesting that Leu 14, BMI 1690, and BMI 1808 are positive for AML leukemic blasts (Additional file 1: Figure S1a). In line with previous studies, these CD45^lo^ AML leukemic blasts from Leu 14, BMI 1690, and BMI 1808 expressed varying levels of CD34, CD38, and CD117 [19–21].

**Table 1:** Immune profile of AML patients’ BM mononuclear cells

		% Relative to human CD45			
Donor	No. of mononuclear cells^a^ injected per NSG pup^b^	CD45^lo^CD34^+^	CD45^lo^CD34^+^CD33^+^CD117^+^	CD45^lo^CD34^−^	CD45^lo^CD34^−^CD33^+^CD117^+^
Leu 14	6.5 × 10^5^	61.4	10.7	35.7	0.1
Leu 29	2.9 × 10^5^	3.1	0.0	8.6	0.0
Leu 32	8.7 × 10^4^	53.0	0.0	27.5	0.0
Leu 33	2.6 × 10^5^	1.1	0.0	15.5	0.0
BMI 1690	5.0 × 10^5^	56.1	1.2	32.7	5.5
BMI 1786	7.9 × 10^5^	72.0	1.0	19.3	0.0
BMI 1808	6.7 × 10^5^	34.7	3.6	50.3	14.1

^a^Refers to CD3-depleted mononuclear cells

^b^Refers to 1–3-day-old NSG newborn

### Samples Leu 14, BMI 1690, and BMI 1808 successfully engrafted in NSG newborn pups via intrahepatic injection

**Fig. 1:**
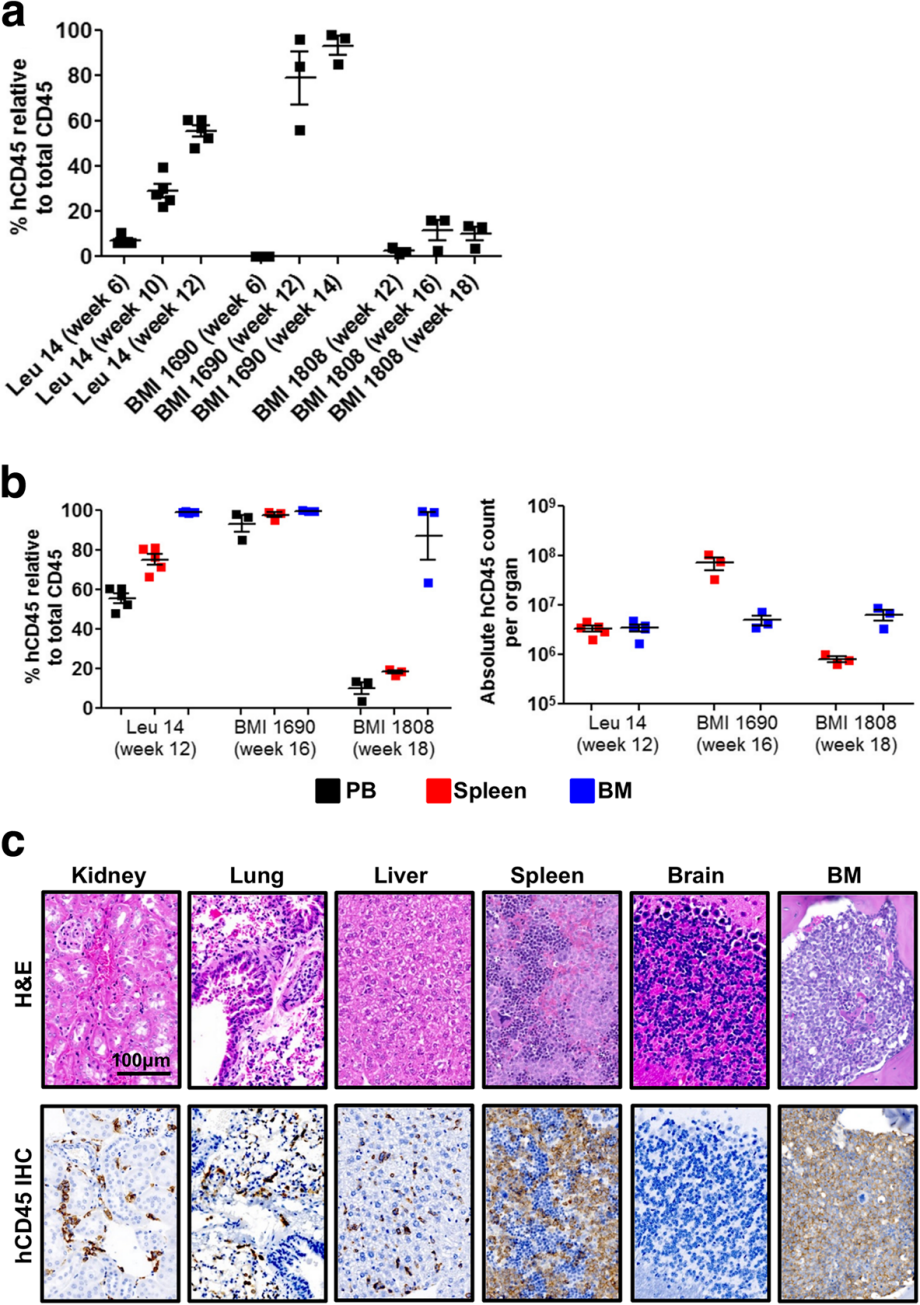
Patient-derived AML cells successfully engraft newborn NSG pups. **a** Level of primary engraftment from good responders (Leu 14, *n* = 5; BMI 1690, *n* = 3; and BMI 1808, *n* = 3). Newborn NSG pups were injected intrahepatically with CD3-depleted mononuclear cells after sublethal irradiation. Level of engraftment was determined at specified week post-engraftment after normalizing human CD45^+^ event numbers by the sum of human CD45^+^ and mouse CD45.1^+^ event numbers in peripheral blood. Data are presented as mean % human CD45^+^ cells relative to total CD45^+^ cells (human CD45^+^ cells + mouse CD45.1^+^ cells) ± SEM. **b** Frequencies and absolute count of human CD45 cells in peripheral blood, spleen, and BM of mice engrafted with Leu 14, BMI 1690, or BMI 1808 at endpoint (week 12–18 post-engraftment). Data are presented as mean % human CD45^+^ cells relative to total CD45^+^ cells ± SEM. **c** Human CD45^+^ cells were detected in major organs except the brain. Engrafted mice were sacrificed at endpoint (week 12–18 post-engraftment) and major organs (kidney, lung, liver, spleen, brain, and BM) collected and stained with H&E and immunohistochemistry against human CD45. Representative images of H&E-stained and human CD45 IHC-stained organs obtained from Leu 14 engrafted mice at week 12 post-engraftment were shown; scale bar: 100 μm

Delivery of fetal liver hematopoietic stem cells via intrahepatic injection into NSG newborn pups is routinely used in the humanization of NSG mice but have yet to be described in AML xenotransplantation [16, 22]. To evaluate the engraftment of AML in NSG mice using intrahepatic injection, CD3-depleted mononuclear cells derived from seven AML patients were injected intrahepatically into sublethally irradiated NSG newborn pups (Fig. 1, Additional file 1: Figure S1b and c, and Table 1). The number of cells injected range from 8.7 × 10^4^–7.9 × 10^5^ per NSG newborn pup (Table 1). The mice were monitored for survival, and peripheral blood was collected submandibularly at 2–4 weeks intervals after week 6 post-engraftment to determine the engraftment level using flow cytometry. The levels of AML engraftment were calculated based on the proportion of human CD45^+^ cells relative to total CD45^+^ cells (human and mouse CD45). Phenotypic analysis revealed that NSG recipient mice injected with Leu 14, BMI 1690, or BMI 1808 were successfully engrafted, with more than 10% AML engraftment detected in the peripheral blood (Fig. 1a and Additional file 1: Figure S1b).

Despite injected with similar number of cells, engraftment of Leu 14 (range 6.0–10.4%) was detected as early as week 6 post-engraftment as compared to BMI 1690 (0.1–0.2%; week 6 post-engraftment) and BMI 1808 (0.1–0.3%; week 8 post-engraftment; data not shown). The frequency of periphery AML cells in Leu 14 and BMI 1690 recipient mice increased with time, with more than 50% AML cells detected at endpoint (week 12–14 post-engraftment; Fig. 1a). In contrast, the frequency of periphery AML cells in BMI 1808 recipient mice persisted around 13–16% at endpoint (week 18 post-engraftment; Fig. 1a).

Consistent with peripheral blood, high frequency of AML cells were detected in spleen (range 16.6–99.1%) and BM (range 63.4–99.9%) of Leu 14, BMI 1690, and BMI 1808 recipient mice at endpoint (Fig. 1b, c, Additional file 1: Figure S1b). These primary engrafted cells expressed similar AML-associated markers to that of primary cells (Additional file 1: Figure S1a). Expression of normal hematopoietic cell markers such as CD3, CD4, CD8, CD19, and CD56 was absent (data not shown). The expression profile of AML cells differs among BM, spleen, and peripheral blood (Additional file 2: Figure S2). In comparison, BM harbors the highest frequency of human CD45^+^ cells, accompanied by the greatest level of CD117 expression, as opposed to spleen and peripheral blood (Additional file 2: Figure S2).

Except for mild enlargement of spleen, no solid tumors were observed in the recipient mice at endpoint (data not shown). Histological assessment by H&E and immunohistochemical staining of human CD45 revealed that AML cells infiltrated into multiple organs such as kidneys, lungs, liver, spleen, and BM, but not the brain (Fig. 1c). Collectively, these results demonstrated that the construction of AML mouse model via intrahepatic injection of patient-derived mononuclear cells into NSG newborn pups was successful and recapitulated human leukemogenesis.

### CD34^+^ AML cells had greater engraftment capacity than CD34^−^ AML cells

**Fig. 2:**
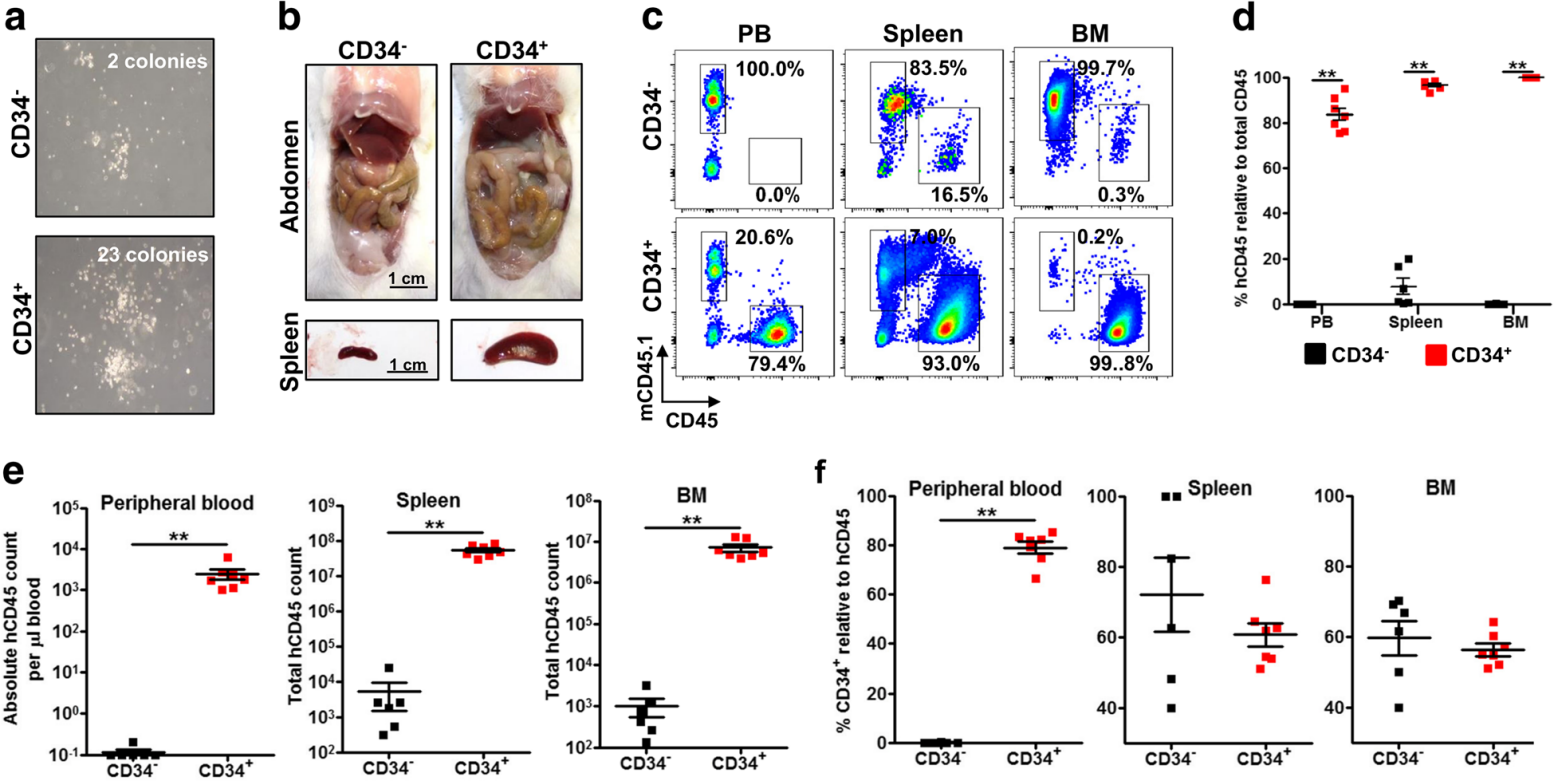
CD34^+^ AML cells have greater proliferative and engraftment capacity than CD34^−^ AML cells. Pooled BM cells and splenocytes from Leu 14 primary engrafted mice were FACs-sorted to CD34^+^ and CD34^−^ fractions. **a** Methocult™ colony-forming unit (CFU) assay of CD34^+^ and CD34^−^ cells. Representative images at 3 weeks post-culture were shown. Colonies were counted microscopically, and the mean number of colonies obtained in duplicate dishes (1 × 10^3^ cells inoculated per dish) was indicated. **b** Evaluation of pathological outcome at week 10 post-engraftment. Representative images of abdomen and spleen from CD34^+^ or CD34^−^ engrafted mice (5 × 10^5^ cell injected per pup) were shown; scale bar: 1 cm. **c** The level of AML engraftment in peripheral blood, spleen, and BM. Representative flow cytometry plots illustrating the frequency of mouse CD45.1^+^ cells and human CD45^+^ cells from CD34^+^ cells (*n* = 7) or CD34^−^ cells (*n* = 6) engrafted mice were shown. **d**, **e** Comparison of AML engraftment in peripheral blood, spleen, and BM between CD34^+^ and CD34^−^ cells engrafted mice at endpoint. Data are presented as mean frequency (**d**) or absolute count (**e**) of human CD45^+^ cells ± SEM. Two-tailed Mann Whitney *U* test; **p < 0.01. **f** Comparison of frequency of total CD34^+^ cells relative to human CD45 cells in peripheral blood, spleen, and BM between CD34^+^ and CD34^−^ cells engrafted mice at endpoint. Data are presented as mean frequencies ± SEM. Two-tailed Mann Whitney *U* test; **p < 0.01

Acute myeloid leukemia initiating cells or stem cells are believed to be restricted in the CD34^+^ compartment [2, 8]. To examine if CD34^+^ cells have greater engraftment capacity in our model, cells pooled from the spleen and BM of primary engrafted mice were sorted for CD34^+^ and CD34^−^ using flow cytometry. Proliferative capacity and colony-forming ability of CD34^+^ cells were assessed in vitro using colony-forming assay. Sorted CD34^+^ fraction gave rise to significantly more colonies (~ 10 fold more) compared to CD34^−^ fraction at 3-weeks post-assay (Fig. 2a), indicating that CD34^+^ cells are more proliferative than CD34^−^ cells. In vivo, secondary NSG recipient mice engrafted with CD34^+^ cells exhibited a more severe pathological outcome compared to mice engrafted with CD34^−^ cells at endpoint (week 10 post-engraftment). While no solid tumors were detected, spleen enlargement and massive accumulation of green soft tissue resembling soft tissue sarcoma with increased vascularization at the trunk, abdomen, limbs, and kidneys were observed in CD34^+^ secondary engrafted NSG mice (Fig. 2b and Additional file 3: Figure S3a). Further histological assessment using H&E and an immunohistochemical panel staining for myeloid sarcoma [23] which included human CD45, myeloperoxidase (MPO), and CD117 revealed massive infiltration of AML cells into multiple organs and confirmed that the soft tissue mass was myeloid sarcoma composed of AML leukemic blasts (Additional file 3: Figure S3b).

Although AML cell engraftment was detected in all secondary NSG recipient mice, engraftment levels were significantly higher in mice engrafted with CD34^+^ than CD34^−^ cells in peripheral blood, spleen, and BM (Fig. 2c–e). The frequency of AML cells was ~ 80 fold, 25 fold, and 100 fold greater in CD34^+^ engrafted mice compared to CD34^−^ engrafted mice in the peripheral blood, spleen, and BM, respectively (Fig. 2c, d). This translated to ~ 18,000 fold, 22,000 fold, and 9000 fold greater in absolute AML count in peripheral blood, spleen, and BM, respectively (Fig. 2e). Phenotypically, except for the frequency of human CD45^+^ cells, there was no significant difference in the frequency of CD34^+^ in the spleen and BM between CD34^−^ and CD34^+^ engrafted mice (Fig. 2f). Taken together, these results indicate that CD34^+^ cell has greater proliferative and engraftment capacity as compared to CD34^−^ cells.

It is not known if AML cells delivered intrahepatically populate peripheral blood, spleen, or BM first. To address this, we sacrificed secondary NSG recipient mice engrafted with CD34^+^ cells early at 4 weeks post-engraftment. Immunophenotypic analysis revealed that AML cells repopulated the BM at greater frequency and absolute count followed by spleen and peripheral blood in descending order (Additional file 4: Figure S4). Taken together, these results suggest that AML cells delivered intrahepatically homed to the site of leukemogenesis (BM) first before progressing to spleen and peripheral blood.

### Engraftment of AML is more efficient in NSG newborn pups as compared to NSG adults

**Fig. 3:**
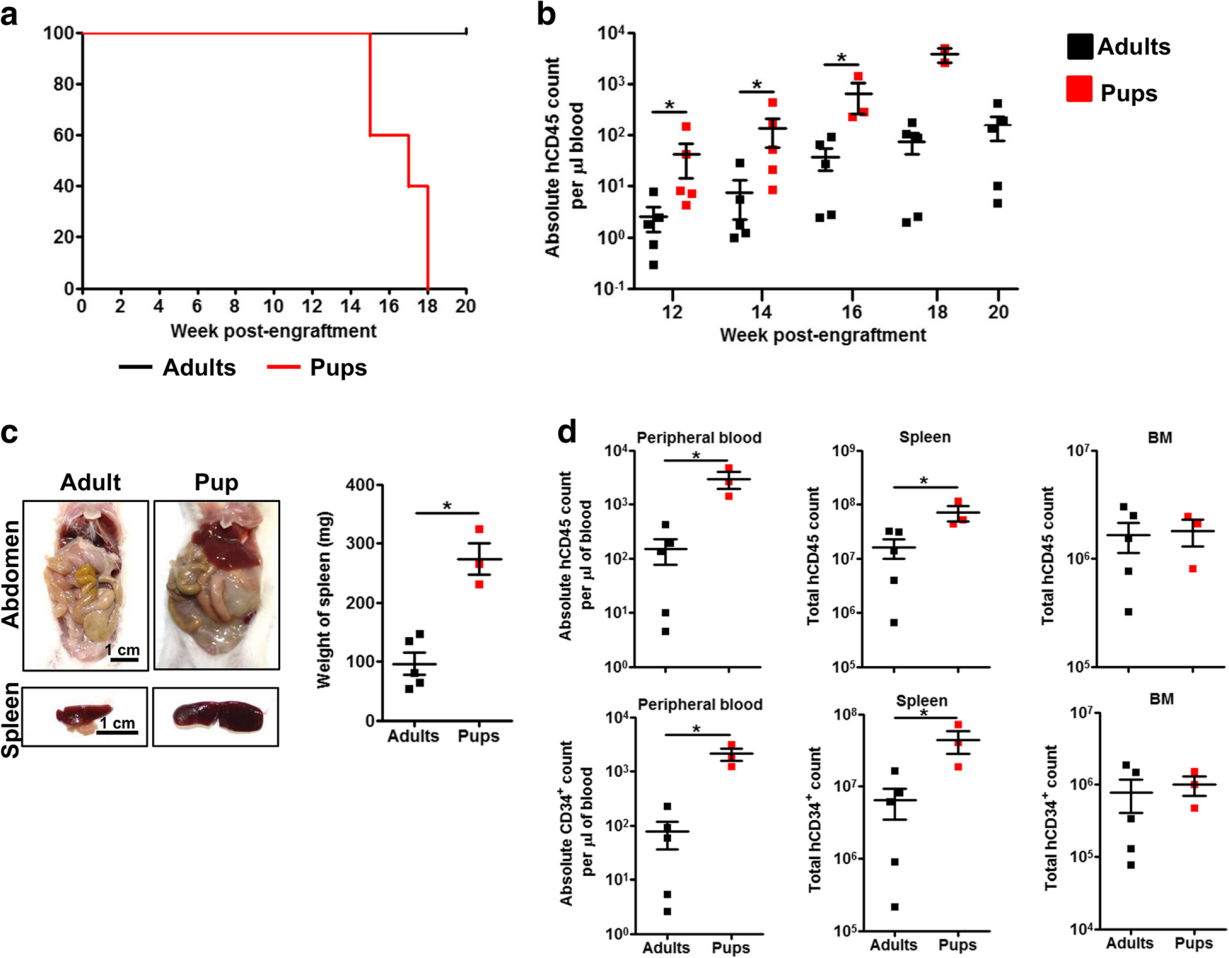
Engraftment of AML cells is more efficient in newborn NSG pups than adult NSG mice. Magnetically sorted CD34^+^ pooled BM cells and splenocytes from secondary engrafted NSG mice were injected intrahepatically in newborn NSG pups (1Gy; *n* = 5) or intravenously in 6-week-old NSG adults (2.5Gy; *n* = 5) after sublethal irradiation (1 × 10^5^ cells per mice). **a** Survival curve of newborn NSG pups and adult NSG mice over 20 weeks. **b** Comparison of longitudinal AML engraftment in peripheral blood between newborn NSG pups and adult NSG mice. Data are presented as mean absolute human CD45^+^ cell count per microliter of blood ± SEM. Two-tailed Mann Whitney *U* test; *p < 0.05. **c** Comparison of pathological outcome and spleen size between engrafted newborn NSG pups and adult NSG mice at endpoint (week 17–20 post-engraftment). Representative images of abdomen and spleen were shown; scale bar: 1 cm. The weight of spleen is presented as mean ± SEM. Two-tailed Mann Whitney *U* test; *p < 0.05. **d** Comparison of absolute count of human CD45^+^ cells (**d**, above) and CD34^+^ cells (**d**, below) in peripheral blood, spleen, and BM between newborn NSG pups and adult NSG mice at endpoint (week 17–18 post-engraftment). Data are presented as mean absolute count ± SEM. Two-tailed Mann Whitney *U* test; *p < 0.05

Next, we compared the engraftment efficiency of AML cells between our model and existing model utilizing NSG adult mice and intravenous injection. Newborn NSG pups and adult mice (6-week-old) were irradiated sublethally before injection with 1 × 10^5^ sorted CD34^+^ cells from secondary engrafted mice via intrahepatic and tail-vein intravenous route, respectively. The mice were monitored for 20 weeks. Engrafted NSG newborn pups began to exhibit weakness at week 15 post-engraftment and were sacrificed when moribund, while all NSG recipient adult mice survived at week 20 post-engraftment (Fig. 3a). Across multiple time points, significantly greater (~ 27 fold greater at endpoint) AML cell engraftment was detected in the peripheral blood of tertiary NSG recipient newborn pups (Fig. 3b) and this translated to pronounced accumulation of abdominal soft tissue mass and significant spleen enlargement as compared to recipient adult mice (Fig. 3c). The absolute number of human CD45^+^ and CD34^+^ AML cells was significantly greater in the peripheral blood and spleen but not in the BM of NSG newborn pups compared to adult mice (Fig. 3d). Phenotypically, except for the frequency of human CD34^+^ cells in the peripheral blood, there was no significant difference in the frequency of human CD45^+^, CD34^+^, or CD117^+^ cells in the peripheral blood, spleen, and BM between NSG recipient newborn pups and adult mice (Additional file 5: Figure S5). Taken together, these results demonstrate that AML engraftment is more efficient in NSG newborn pups as compared to NSG adult mice.

### CD117^+^ AML cell exhibited enhanced proliferative and engraftment capacity

**Fig. 4:**
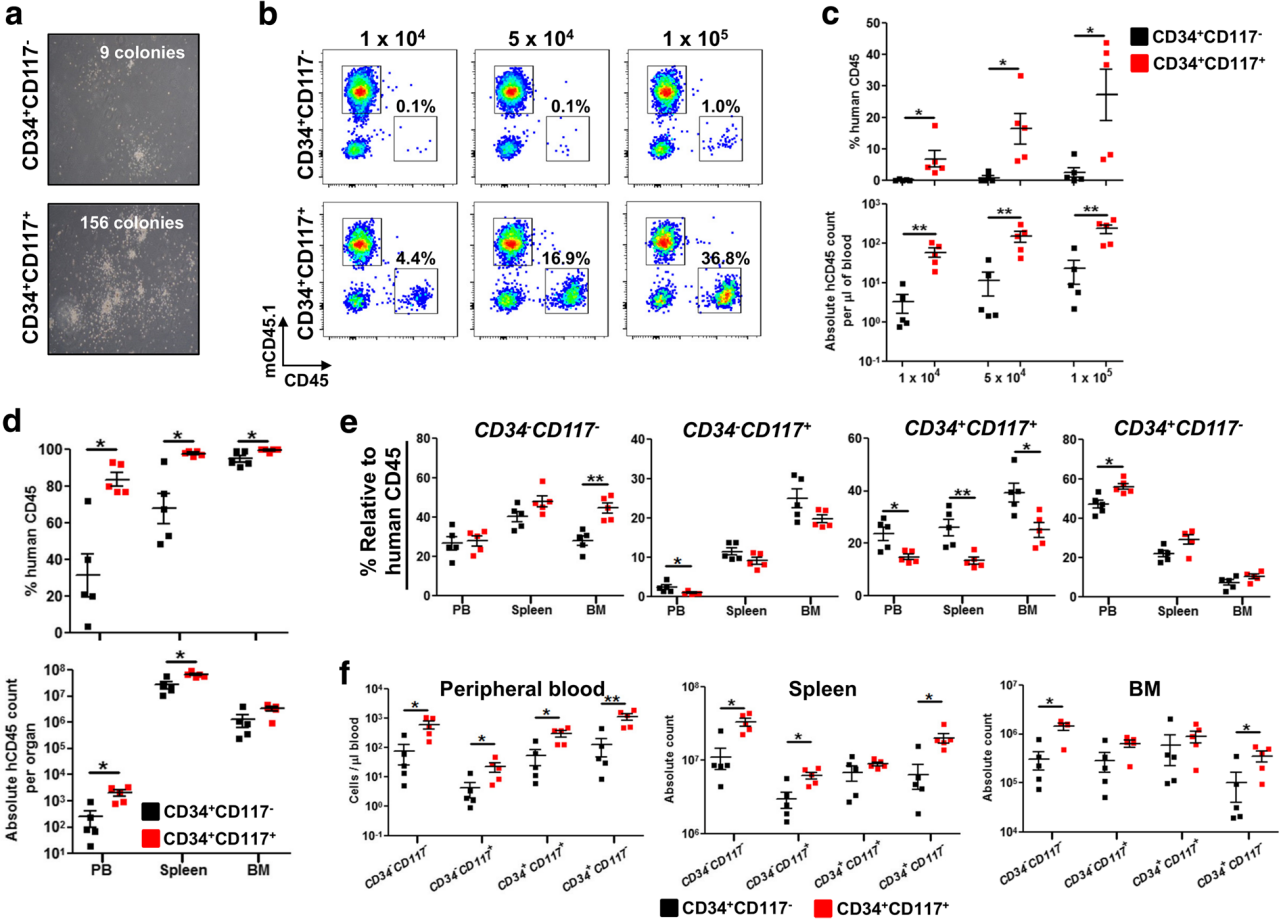
CD117 expression enhances AML engraftment but is not essential to initiate leukemia. Pooled BM cells and splenocytes from primary engrafted NSG mice were magnetically sorted to CD34^+^CD117^+^ and CD34^+^CD117^−^ AML cells. **a** Methocult™ colony-forming unit (CFU) assay. Representative images at 3 weeks post-culture were shown. Colonies were counted microscopically and the mean number of colonies obtained in duplicate dishes (1 × 10^4^ cells inoculated per dish) was indicated. **b**, **c** Vary number of either CD34^+^CD117^−^ or CD34^+^CD117^+^ AML cells (1 × 10^4^, 5 × 10^4^ and 1 × 10^5^) were injected intrahepatically after sublethal irradiation in newborn NSG pups (*n* = 5 per group). Representative flow cytometry plots (**b**), frequency (**c**, above), and absolute count (**c**, below) of AML engraftment in peripheral blood at week 12 post-engraftment. Data are presented as mean frequency or absolute count of human CD45^+^ cells ± SEM. Two-tailed Mann Whitney *U* test; *p < 0.05, **p < 0.01. **d** Comparison of AML engraftment at endpoint (week 16 post-engraftment) in peripheral blood, spleen, and BM between newborn NSG pups engrafted with 1 × 10^5^ of CD34^+^CD117^+^ and CD34^+^CD117^−^ AML cells. Data are presented as mean frequency (**d**, above) or absolute count (**d**, below) of human CD45^+^ cells ± SEM. Two-tailed Mann Whitney *U* test; *p < 0.05. **e**, **f** Immunophenotypic analysis of CD34^+^CD117^+^ and CD34^+^CD117^−^ engrafted recipient mice. Frequency **e** and absolute count **f** of *CD34* ^*−*^ *CD117* ^*−*^, *CD34* ^*−*^ *CD117* ^*+*^, *CD34* ^*+*^ *CD117* ^*+*^, and *CD34* ^*+*^ *CD117* ^*−*^ subsets in peripheral blood, spleen, and BM at week 16 post-engraftment. Data are presented as mean frequency of human CD45^+^ cells or absolute count ± SEM. Two-tailed Mann Whitney *U* test; *p < 0.05, **p < 0.01

Stem cell factor receptor (CD117) and its ligand (stem cell factor) have been implicated to play a key role in hematopoiesis and leukemogenesis [24, 25]. Given that CD117 is expressed on leukemic blasts of Leu 14, BMI 1690, and BMI 1808 among the seven AML patients (Additional file 1: Figure S1), we hypothesized that CD117 is essential for successful engraftment (Fig. 1 and Additional file 2: Figure S2). To test this notion, cells pooled from the spleen and BM of primary engrafted mice were magnetically sorted to CD34^+^CD117^+^ and CD34^+^CD117^−^ fractions and evaluated for their proliferative and engraftment capacity (Fig. 4). Colony forming assay in vitro demonstrated that CD34^+^CD117^+^ cells were more proliferative and formed significantly more colonies than CD34^+^CD117^−^ cells after 3 weeks (Fig. 4a). Although peripheral blood engraftment was detected in both CD34^+^CD117^+^ and CD34^+^CD117^−^ NSG recipient mice at week 12 post-engraftment, the frequency and absolute count of AML cells were significantly greater in CD34^+^CD117^+^ NSG recipient mice in a dose-dependent manner (Fig. 4b, c). At week 16 post-engraftment, NSG recipient mice engrafted with 1 × 10^5^ CD34^+^CD117^+^ or CD34^+^CD117^−^ cells were sacrificed to evaluate their engraftment levels and pathological outcomes. Significantly greater engraftment was observed in the peripheral blood, spleen, and BM of CD34^+^CD117^+^ NSG recipient mice (Fig. 4d). Although either CD34^+^CD117^+^ or CD34^+^CD117^−^ cells were used for engraftment, CD34^−^CD117^−^, CD34^−^CD117^+^, CD34^+^CD117^+^, and CD34^+^CD117^−^ subsets were present in all NSG recipient mice (Fig. 4e). Interestingly, significantly greater frequency of CD34^+^CD117^+^ subset (but not absolute count) was detected in mice engrafted with CD34^+^CD117^−^ (Fig. 4e, f). Despite significant increase in engraftment, CD34^+^CD117^+^ NSG recipient mice did not exhibit more severe pathological outcome than CD34^+^CD117^−^ NSG recipient mice (data not shown). Collectively, these results indicated that AML initiating cells are enriched but do not reside exclusively in the CD117^+^ fraction.

### Sorafenib and Regorafenib treatment suppressed AML cells engraftment

**Fig. 5:**
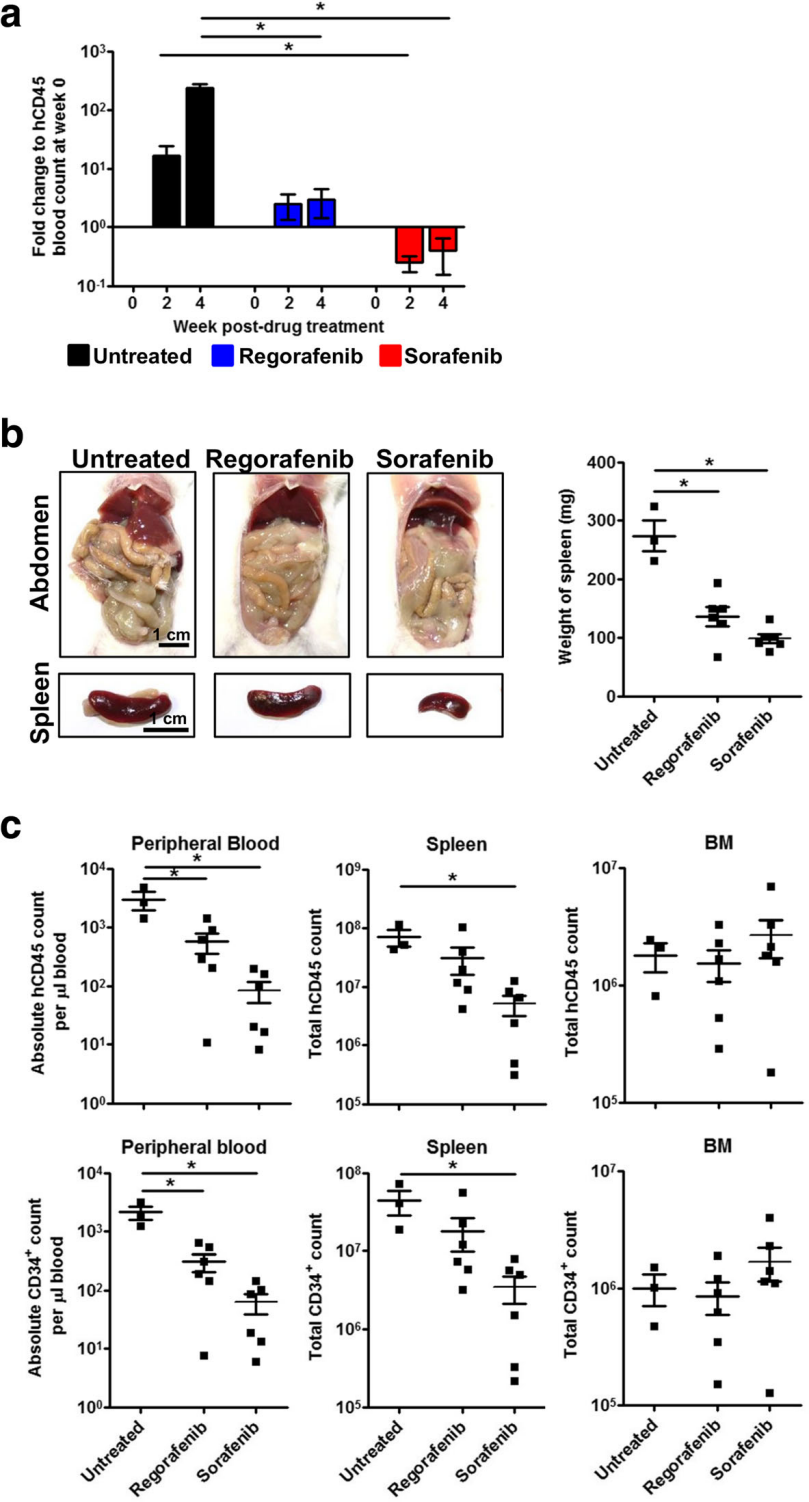
Sorafenib and Regorafenib treatment suppressed AML engraftment in vivo. Magnetically sorted CD34^+^ pooled BM cells and splenocytes from secondary engrafted NSG mice were injected intrahepatically in newborn NSG pups after sublethal irradiation (1 × 10^5^ cells per pup). Successfully engrafted mice with more than 30 human CD45^+^ cells per microliter of blood (between week 12 to 16 post-engraftment) were randomly assigned to either untreated (*n* = 3), Regorafenib (*n* = 6; 5 mg/kg body weight; gavage-fed once daily) or Sorafenib (*n* = 6; 10 mg/kg body weight; gavage-fed once daily) treatment groups and monitored for 1 month. **a** Longitudinal effect of Regorafenib and Sorafenib treatment on peripheral blood engraftment at week 0, 2, and 4 post-drug treatment. Change in AML engraftment for each group at each time point is expressed as fold change relative to the absolute human CD45^+^ count per microliter of blood at week 0 post-drug treatment. Data are presented as mean fold change ± SEM. Two-tailed Mann Whitney *U* test; *p < 0.05. **b** Soft tissue sarcoma and reduced spleen size were observed in Regorafenib- or Sorafenib-treated mice. Representative images of abdomen and spleen was shown; scale bar: 1 cm. Weight of spleen are presented as mean ± SEM. Two-tailed Mann Whitney *U* test; *p < 0.05. **c** Comparison of the absolute count of human CD45^+^ cells (**c**, above) and CD34^+^ cells (**c**, below) in peripheral blood, spleen, and BM between different treatment groups after 4 weeks post-drug treatment. Data are presented as mean absolute count ± SEM. Two-tailed Mann Whitney *U* test; *p < 0.05

Sorafenib (Nexavar®) and Regorafenib (Stivarga®) are FDA-approved multi-kinase inhibitors that have shown effective anti-tumor activity in patients with solid tumors [26, 27]. More recently, the potential usefulness of Sorafenib and Regorafenib in AML treatment has emerged in numerous in vitro, pre-clinical studies and phase I/II clinical trials [28–33]. To evaluate the potential use of our model as a pre-clinical tool for therapeutics assessment in vivo, successfully engrafted NSG recipient mice were gavage-fed a daily dose of Sorafenib (10 mg/kg body weight) or Regorafenib (5 mg/kg body weight) and monitored for 1 month. Peripheral blood engraftment was significantly reduced in Sorafenib- and Regorafenib-treated mice compared to untreated mice at week 2 and 4 post-treatment (Fig. 5a). The fold change of reduction was more drastic in Sorafenib treatment, as the level of engraftment after 2 weeks of treatment fell below the engraftment level before treatment. In contrast, Regorafenib treatment suppressed the rate of increase in engraftment level (Fig. 5a). At the endpoint, Sorafenib and Regorafenib treatment did not result in complete resolution of myeloid sarcoma. The extent of myeloid sarcoma was reduced, accompanied by significant reduction in spleen size (Fig. 5b). While the frequency of CD34^+^ and CD117^+^ cells remain unchanged between untreated and treated mice (Additional file 6: Figure S6), significant reduction of absolute AML count was observed in the peripheral blood, spleen but not BM upon Sorafenib and Regorafenib treatment (Fig. 5c). This suggests that AML cells in the peripheral blood and spleen are sensitive to Sorafenib and Regorafenib, whereas those in the BM are protected from the drugs. Overall, the favorable response of Sorafenib and Regorafenib in AML treatment can be recapitulated in our model.

## Discussion

The availability of an in vivo model for human AML is attractive for the understanding of AML biology and for the development of new therapeutic strategies. Due to its prolonged lifespan and enhanced engraftment capacity, xenotransplantation of patient-derived AML cells in NSG mice has long been considered the gold standard for evaluating human hematologic malignancies. However, existing models are not without limitations. As shown by others and by us in this study, engraftment of AML cells is less efficient and unpredictable when transplanted in NSG adult recipient mice [2, 11]. The influence of age in engraftment efficiency was addressed by Ishikawa et al. in 2007, where engraftment efficiency was shown to be enhanced when AML cells were transplanted into newborn NSG pups via craniofacial intravenous injection [2]. Despite the improvement and usefulness, this model is not commonly adopted by the scientific community in the past decade as it is technically difficult to construct.

In contrast, our approach which uses intrahepatic injection is widely-accepted and routinely used in the humanization of NSG mice [16, 22]. Technically, intrahepatic injection of AML cells in newborn NSG pups is relatively easy as compared to neonatal intravenous injection or intrahepatic injection in adult NSG mice, as the liver is visually obvious through the skin of newborn pups and has a large surface area for injection. In this model, we consistently observed high levels of engraftment (> 10%) in the peripheral blood, spleen, and BM in all samples exhibiting CD45^lo^CD33^+^ AML leukemic blast phenotypes (3/3). The engrafted AML cells retained the phenotype of primary cells. The engraftment levels and phenotypes persisted in secondary and tertiary recipients and were not altered by multiple passages in mice. Pathological features of human AML including myeloid sarcoma, spleen enlargement, and infiltration of leukemic cells into circulation and tissues were recapitulated in our model. Furthermore, transplantation dose as low as 1 × 10^4^ CD34^+^ cells is sufficient for the construction of this AML model. Mice engrafted with 1 × 10^5^ CD34^+^ cells can expand in vivo to yield large number of AML cells (10^6^–10^8^) cells after 16 weeks. Through serial transplantation, the recipient mice can potentially provide an unlimited source of AML cells repetitively for direct experimental use, downstream molecular analysis, and ex vivo genetic manipulations. Taken together, these results demonstrate the robustness and specificity of our model with potential for long-term characterization of engrafted patient cells.

The ability to detect circulating AML cells in the peripheral blood allows examination of AML cells in a single recipient mouse across multiple time points. To take advantage of this, attempts were made to identify an immunophenotype that characterized leukemic stem cells which are believed to influence engraftment potential in NSG recipient mice and are responsible for disease resistance or relapse in patients [34]. Consistent with a recent report [15], we have demonstrated, through both in vitro and in vivo studies, that leukemic stem cells are enriched in the CD34^+^ population and in particular CD34^+^CD117^+^ fraction, while CD34^+^CD117^−^ fraction is more mature and less potent in proliferation. Interestingly, CD34^+^CD117^−^ engrafted mice gave rise to both CD117^−^ and CD117^+^ cells; therefore, it is not clear if leukemogenesis and pathological outcomes observed in CD34^+^CD117^−^ engrafted mice are driven by CD117^−^ or CD117^+^ cells. Given the heterogeneity and plasticity of leukemic stem cells, these results raise the possibility that the engrafted CD34^+^CD117^−^ cells can de-differentiate to give rise to CD34^+^CD117^+^ cells and acquire an immature, stem cell-like property to drive the disease progression [12, 35, 36].

Although it is generally accepted that leukemic stem cells are phenotypically characterized as CD34^+^CD38^−^ [2, 8, 34], increasing evidence from various groups have challenged this notion and have demonstrated that leukemic stem cells also exist in the CD34^+^CD38^+^ fraction and CD34^−^ subpopulation [12, 14, 37–39]. It is possible that the leukemic stem cell activity is mediated by the CD34^+^CD38^+^ population as majority of the CD34^+^ cells from Leu 14, BMI 1690, and BMI 1808 patients express high levels of CD38.

Global gene expression profiling of AML cells before and after transplantation in mice might be useful to ascertain if the observed differential phenotype is due to an outgrowth of a subclone or because AML cells acquired a different differentiation pattern in mice as opposed to patients [40]. This does not imply that xenotransplantation in NSG mice is unstable, but rather it underscores the plasticity of leukemic stem cells, such that their commitment to certain fate(s) can be multidirectional or reversible, depending on the intrinsic and extrinsic signals [36]. Clonal evolution of AML has been observed previously in patients during relapse and xenograft models during serial transplantations [40–42]; however, the mechanism underlying clonal evolution is not known. With a larger patient cohort, these dimensions can be further investigated using our model.

In addition, the discrepancies in engraftment potential as shown by the “low engrafters” (Leu 32 and Leu 1786) despite the presence of CD45^lo^ leukemic blasts can possibly be a reflection of their in vivo proliferative ability, or alternatively, an indication of prognosis. It was reported in previous studies that AML cells from patients with poor prognosis features such as the presence of FLT3 mutations, high white blood cell count at diagnosis, or chromosomal rearrangements would tend to engraft more efficiently in mice than AML cells isolated from patients with good prognostic features [9, 40, 43]. Intrahepatic delivery of these “low engrafters” into neonatal NSGS mouse strain can also be explored in future studies, as it was demonstrated that constitutive expression of human cytokines (SCF, GM-CSF, and IL-3) in NSGS mice improved engraftment efficiency of “low engrafters” [15].

Chemotherapy drug resistance mediated by BM microenvironment is increasingly recognized as a major obstacle to the treatment of AML [2, 44]. The BM microenvironment, which is rich in growth factors, cytokines, and stromal cells, provides a permissive environment for leukemogenesis and also contributes to chemotherapy resistance through mechanisms involving growth factors and cell-cell interaction [45]. Adhesion of leukemic blasts to marrow stromal cells and fibronectin via molecules, such as CD117 and CXCR4, or to osteoblast-rich areas has been shown to protect AML leukemic blasts from drug-induced apoptosis [2, 46, 47]. Thus, it is not surprising to observe Sorafenib- and Regorafenib-induced apoptotic, anti-proliferative, and anti-angiogenic effects on leukemic blasts in the periphery and spleen but not BM [48, 49]. Our work suggests that future therapeutic strategy should consider drug design that directly targets the leukemic blasts in the BM. Alternatively, Sorafenib/Regorafenib can be combined with small molecule inhibitor (e.g., AMD3100; CXCR4 inhibitor or CD117 inhibitor) that disrupts the leukemic blasts-BM interactions and mobilizes leukemic blasts to the periphery, thereby sensitizing them to the cytotoxic effects induced by Sorafenib and Regorafenib [10]. This new model can provide a pre-clinical platform for the testing of the combined therapies.

## Conclusions

In conclusion, this study describes an improved patient-derived AML murine model that is robust and easy-to-construct. It recapitulates many aspects of human AML and, therefore, has the potential to be reliably employed for pre-clinical studies.

## Additional files

Additional file 1: Figure S1. Immune profile of BM mononuclear cells from AML patients. **a** Mononuclear cells isolated from AML patients were immunolabeled with human CD45, CD34, CD38, CD33, and CD117 and analyzed using flow cytometry. Gating using CD45 and CD34 showed three subsets indicating of (i) CD45^hi^CD34^−^ non-blast cells, (ii) CD45^lo^CD34^−^ blast cells, and (iii) CD45^lo^CD34^+^ blast cells. Expression of CD38, CD33, and CD117 for each subset was shown. Frequency of CD33^+^CD117^+^ relative to total human CD45^+^ cells in each subset was shown. **b** Level of primary engraftment from poor responders. Newborn NSG pups were injected intrahepatically with 8.7 × 10^4^–7.9 × 10^5^ cells after sublethal irradiation. Level of engraftment in peripheral blood was determined at specified weeks post-engraftment and in spleen and BM at endpoint using event number of human CD45^+^ cells divided by the sum of human CD45^+^ cells and mouse CD45.1^+^ cells. Data are presented as mean % human CD45^+^ cells relative to total CD45^+^ cells ± SEM. (TIFF 3265 kb)

Additional file 2: Figure S2. Immune profile of AML engrafted NSG mice at endpoint. Peripheral blood obtained from NSG recipient mice engrafted with Leu 14, BMI 1690, and BMI 1808 were immunolabeled with human CD45, CD34, CD38, CD33, and CD117 and analyzed using flow cytometry at endpoint. Frequency of subsets is presented as % relative to total human CD45^+^ cells. (TIFF 2730 kb)

Additional file 3: Figure S3. AML mice developed myeloid sarcoma. **a** Representative images of multiple organs from CD34^+^ engrafted mice were shown (scale bar: 1 cm) and **b** analyzed using H&E and immunohistochemical stain for human CD45, CD117, and MPO. Representative images of multiple organs were shown; scale bar: 1 cm or 100 μm as indicated. (TIFF 13606 kb)

Additional file 4: Figure S4. Engraftment of AML cells is highest in the BM at week 4 post-engraftment. Magnetically sorted CD34^+^ pooled BM cells and splenocytes from primary engrafted NSG mice were injected intrahepatically in NSG newborn pups (*n* = 4) after sublethal irradiation (1 × 10^5^ cells per pup). **a** Frequencies of mouse CD45.1^+^ cells and human CD45^+^ cells in peripheral blood, BM, and spleen were determined at week 4 post-engraftment. Frequency of human CD45^+^ cells and mouse CD45.1^+^ cells are calculated by normalizing the event number of human CD45^+^ cells or mouse CD45.1^+^ cells over the sum of human CD45^+^ cells and mouse CD45.1^+^ cells event numbers. **b** Frequency and c absolute count of human CD45^+^ cells in peripheral blood, spleen, and BM. Data are presented as mean frequencies or absolute count per organ ± SEM. Two-tailed Mann Whitney *U* test; *; *p* < 0.05. (TIFF 3335 kb)

Additional file 5: Figure S5. Frequency of CD34^+^ AML cells is greater in newborn NSG pups than adult NSG mice. Magnetically sorted CD34^+^ pooled BM cells and splenocytes from secondary engrafted NSG mice were injected intrahepatically in newborn NSG pups (1Gy; *n* = 5) or intravenously in 6-week-old NSG adults (2.5Gy; *n* = 5) after sublethal irradiation (1 × 10^5^ cells per adult or pup). **a** Representative flow cytometry plots illustrating the expression of CD34 and CD117 in human CD45 cells in peripheral blood, spleen, and BM of NSG pups and adult NSG mice at endpoint. **b**, **c**, and **d** Comparison of frequency of human CD45 relative to total CD45 **b**, total CD34^+^ **c**, and CD34^+^CD117^+^ **d** cells relative to human CD45 cells in peripheral blood, spleen, and BM between newborn NSG pups and adult NSG mice at endpoint (week 17–20 post-engraftment). Data are presented as mean frequencies ± SEM. Two-tailed Mann Whitney *U* test; *; *p* < 0.05. (TIFF 2359 kb)

Additional file 6: Figure S6. Sorafenib and Regorafenib treatment has no impact on the frequency of CD34^+^ and CD34^+^CD117^+^ AML cells in mice. Magnetically sorted CD34^+^ pooled BM cells and splenocytes from secondary-engrafted NSG mice were injected intrahepatically in newborn NSG pups after sublethal irradiation (1 × 10^5^ cells per pup). Successfully engrafted mice with more than 30 human CD45^+^ cells per microliter of blood (between week 12 to 16 post-engraftment) were randomly assigned to either untreated (*n* = 3), Regorafenib (*n* = 6; 5 mg/kg body weight; gavage-fed once daily), or Sorafenib (*n* = 6; 10 mg/kg body weight; gavage-fed once daily) treatment groups and monitored for 1 month. **a** Representative flow cytometry plots illustrating the expression of CD34 and CD117 in human CD45 cells. **b** Comparison of the frequencies of total CD34^+^ (B, above) and CD34^+^CD117^+^ (B, below) cells relative to human CD45 cells in peripheral blood, spleen, and BM of different treatment groups after 4 weeks post-drug treatment. Data are presented as mean frequencies ± SEM. (TIFF 1403 kb)

## Abbreviations

AML: Acute myeloid leukemia
BM: Bone marrow
CFU: Colony-forming unit
FAB: French-American-British
H&E: Hematoxylin & Eosin
MPO: Myeloperoxidase
NSG: NOD*-scid Il2rγ* ^*null*^
NOD: Non-obese diabetic
PBS: Phosphate buffered saline
RBC: Red blood cells
SCID: Severe combined immunodeficient
SEM: Standard error of mean
