# Supplementary figures and images for: An improved pre-clinical patient-derived liquid xenograft mouse model for acute myeloid leukemia
Source: J Hematol Oncol. 2017 Oct 6;10:162. doi: 10.1186/s13045-017-0532-x (PMC5639594; doi:10.1186/s13045-017-0532-x)

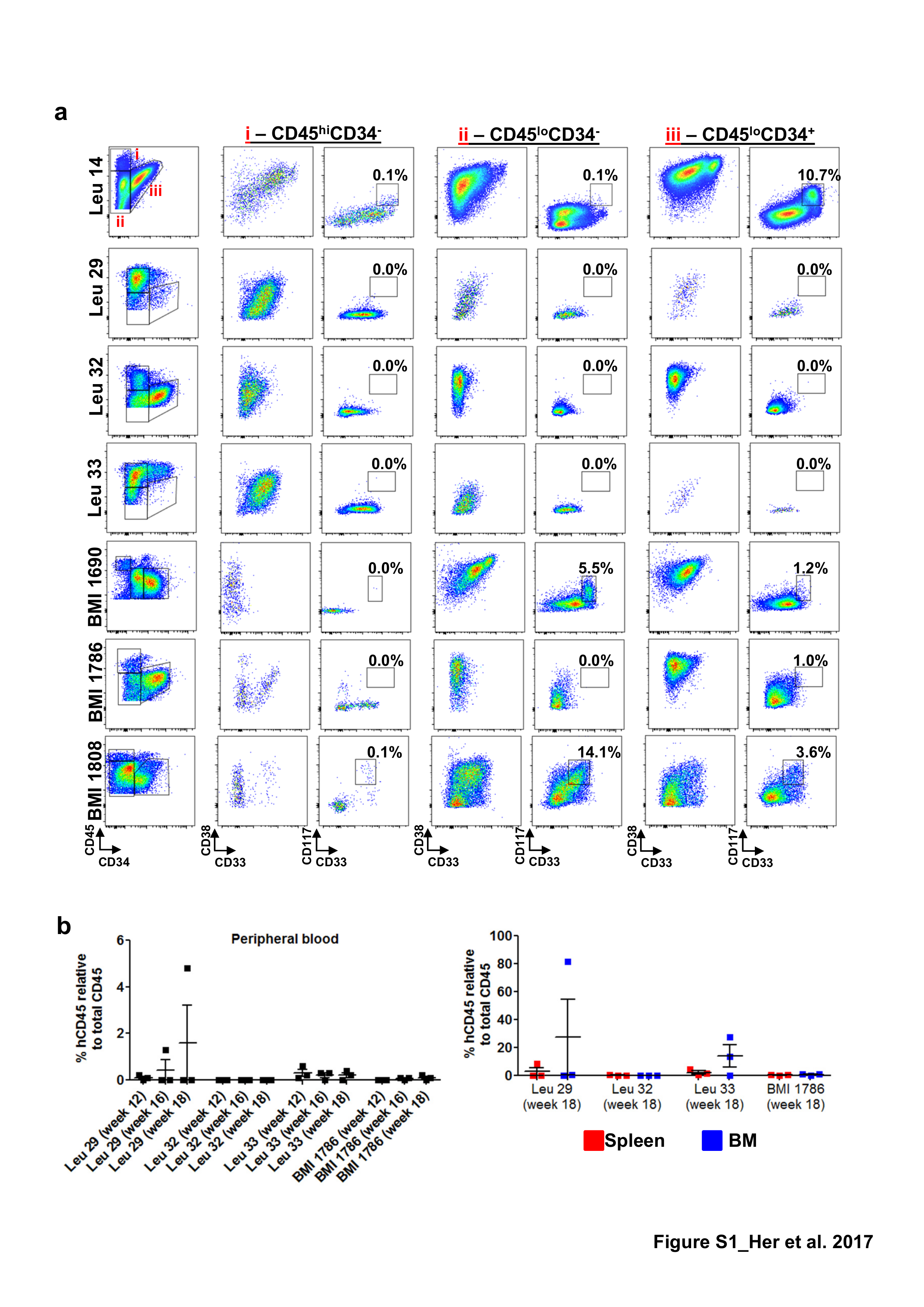

Supplement: Supplementary file 1 — Immune profile of BM mononuclear cells from AML patients. a Mononuclear cells isolated from AML patients were immunolabeled with human CD45, CD34, CD38, CD33, and CD117 and analyzed using flow cytometry. Gating using CD45 and CD34 showed three subsets indicating of (i) CD45hiCD34− non-blast cells, (ii) CD45loCD34− blast cells, and (iii) CD45loCD34+ blast cells. Expression of CD38, CD33, and CD117 for each subset was shown. Frequency of CD33+CD117+ relative to total human CD45+ cells in each subset was shown. b Level of primary engraftment from poor responders. Newborn NSG pups were injected intrahepatically with 8.7 × 10^4^–7.9 × 10^5^ cells after sublethal irradiation. Level of engraftment in peripheral blood was determined at specified weeks post-engraftment and in spleen and BM at endpoint using event number of human CD45+ cells divided by the sum of human CD45+ cells and mouse CD45.1+ cells. Data are presented as mean % human CD45+ cells relative to total CD45+ cells ± SEM. (TIFF 3265 kb) [file 13045_2017_532_MOESM1_ESM.tiff]

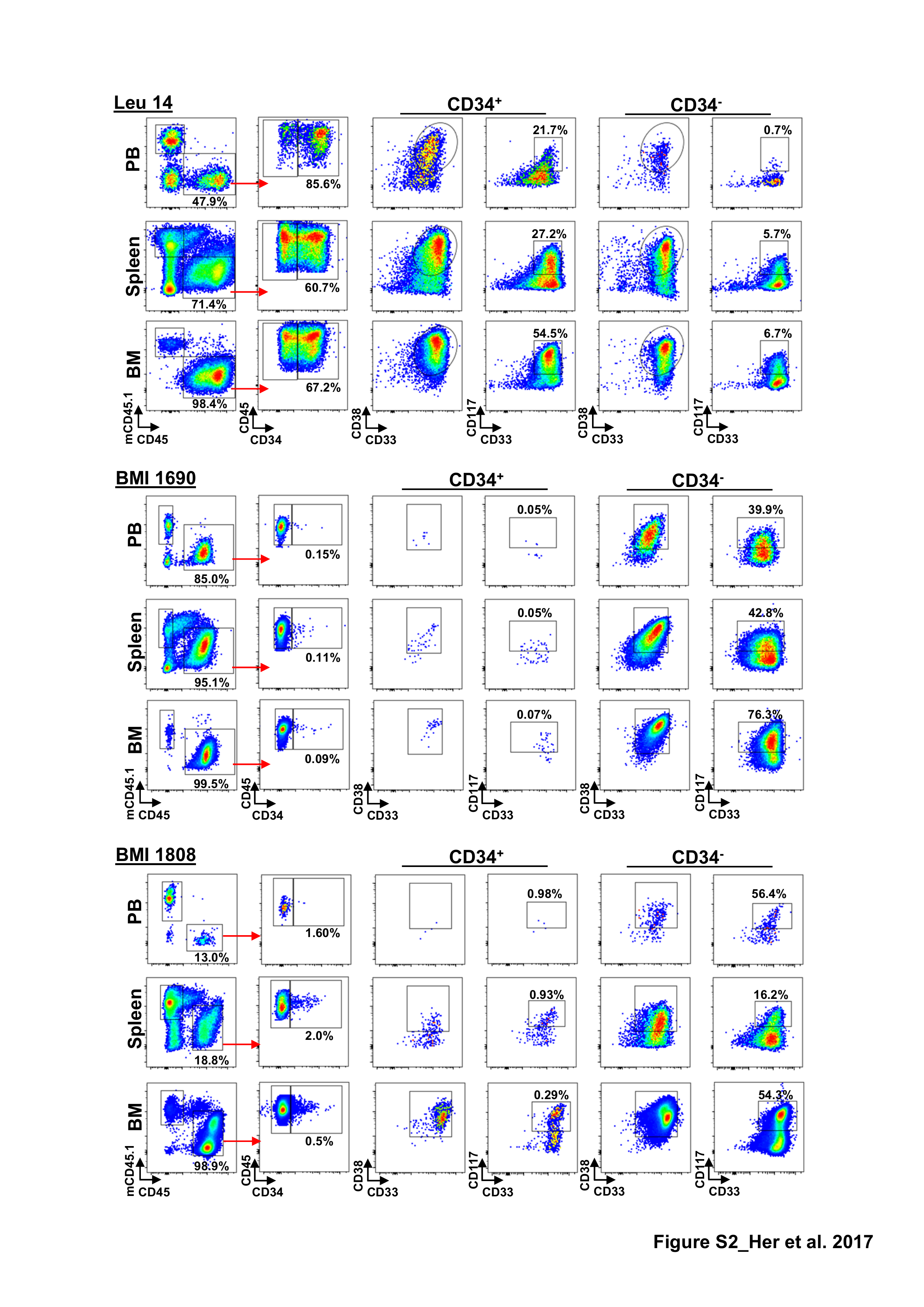

Supplement: Supplementary file 2 — Immune profile of AML engrafted NSG mice at endpoint. Peripheral blood obtained from NSG recipient mice engrafted with Leu 14, BMI 1690, and BMI 1808 were immunolabeled with human CD45, CD34, CD38, CD33, and CD117 and analyzed using flow cytometry at endpoint. Frequency of subsets is presented as % relative to total human CD45+ cells. (TIFF 2730 kb) [file 13045_2017_532_MOESM2_ESM.tiff]

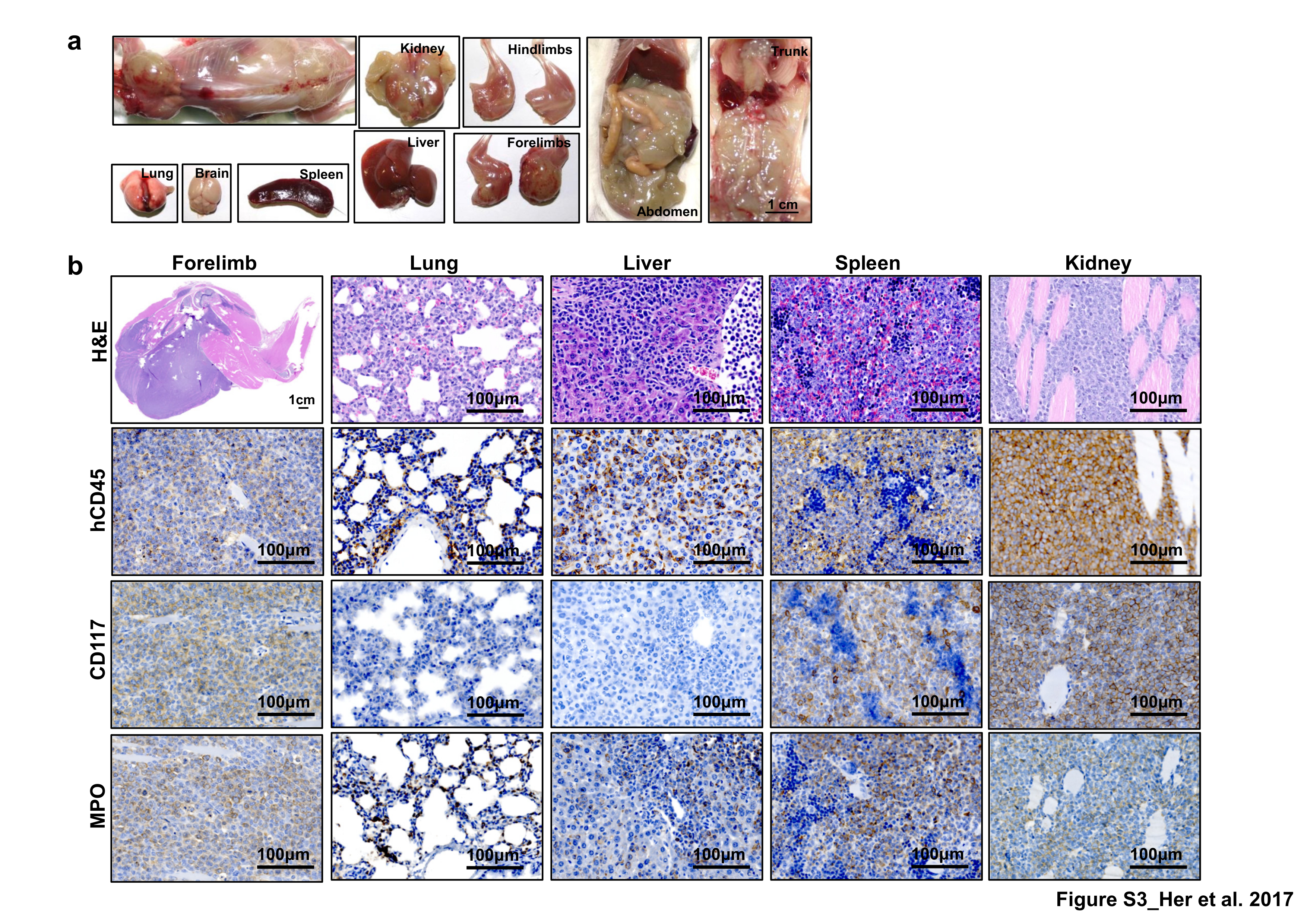

Supplement: Supplementary file 3 — AML mice developed myeloid sarcoma. a Representative images of multiple organs from CD34+ engrafted mice were shown (scale bar: 1 cm) and b analyzed using H&E and immunohistochemical stain for human CD45, CD117, and MPO. Representative images of multiple organs were shown; scale bar: 1 cm or 100 μm as indicated. (TIFF 13606 kb) [file 13045_2017_532_MOESM3_ESM.tiff]

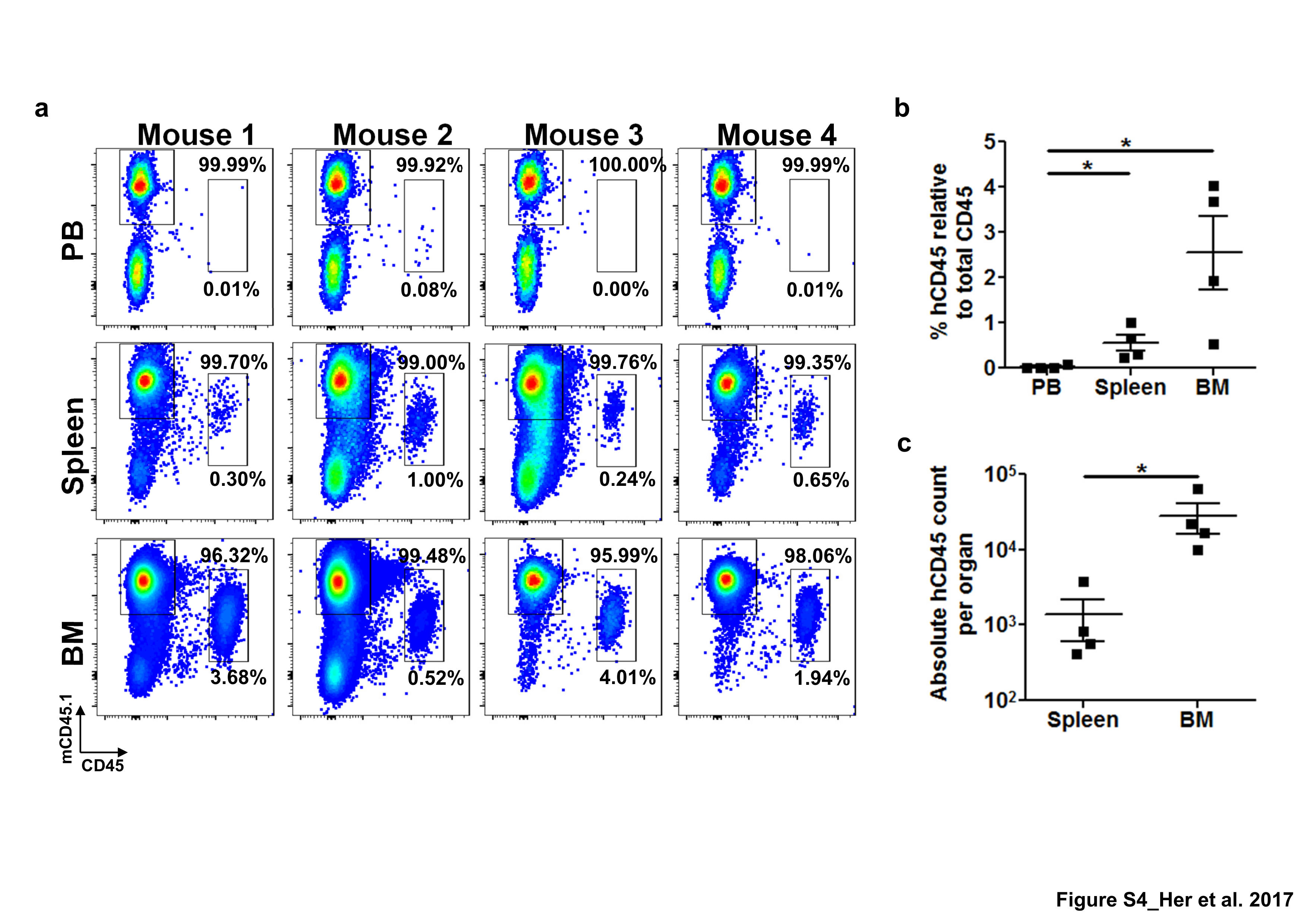

Supplement: Supplementary file 4 — Engraftment of AML cells is highest in the BM at week 4 post-engraftment. Magnetically sorted CD34+ pooled BM cells and splenocytes from primary engrafted NSG mice were injected intrahepatically in NSG newborn pups (n = 4) after sublethal irradiation (1 × 10^5^ cells per pup). a Frequencies of mouse CD45.1+ cells and human CD45+ cells in peripheral blood, BM, and spleen were determined at week 4 post-engraftment. Frequency of human CD45+ cells and mouse CD45.1+ cells are calculated by normalizing the event number of human CD45+ cells or mouse CD45.1+ cells over the sum of human CD45+ cells and mouse CD45.1+ cells event numbers. b Frequency and c absolute count of human CD45+ cells in peripheral blood, spleen, and BM. Data are presented as mean frequencies or absolute count per organ ± SEM. Two-tailed Mann Whitney U test; *; p < 0.05. (TIFF 3335 kb) [file 13045_2017_532_MOESM4_ESM.tiff]

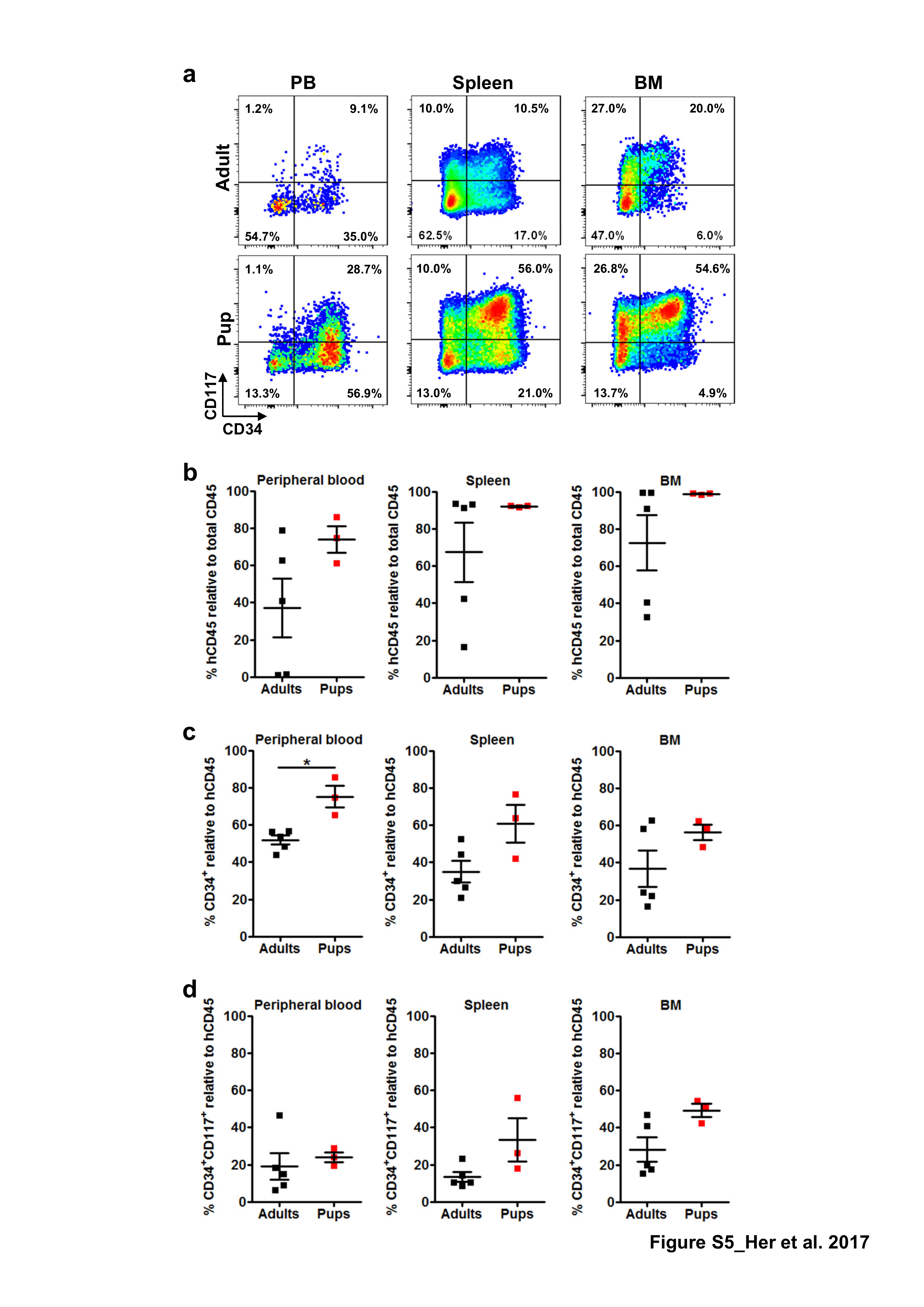

Supplement: Supplementary file 5 — Frequency of CD34+ AML cells is greater in newborn NSG pups than adult NSG mice. Magnetically sorted CD34+ pooled BM cells and splenocytes from secondary engrafted NSG mice were injected intrahepatically in newborn NSG pups (1Gy; n = 5) or intravenously in 6-week-old NSG adults (2.5Gy; n = 5) after sublethal irradiation (1 × 10^5^ cells per adult or pup). a Representative flow cytometry plots illustrating the expression of CD34 and CD117 in human CD45 cells in peripheral blood, spleen, and BM of NSG pups and adult NSG mice at endpoint. b, c, and d Comparison of frequency of human CD45 relative to total CD45 b, total CD34+ c, and CD34+CD117+ d cells relative to human CD45 cells in peripheral blood, spleen, and BM between newborn NSG pups and adult NSG mice at endpoint (week 17–20 post-engraftment). Data are presented as mean frequencies ± SEM. Two-tailed Mann Whitney U test; *; p < 0.05. (TIFF 2359 kb) [file 13045_2017_532_MOESM5_ESM.tiff]

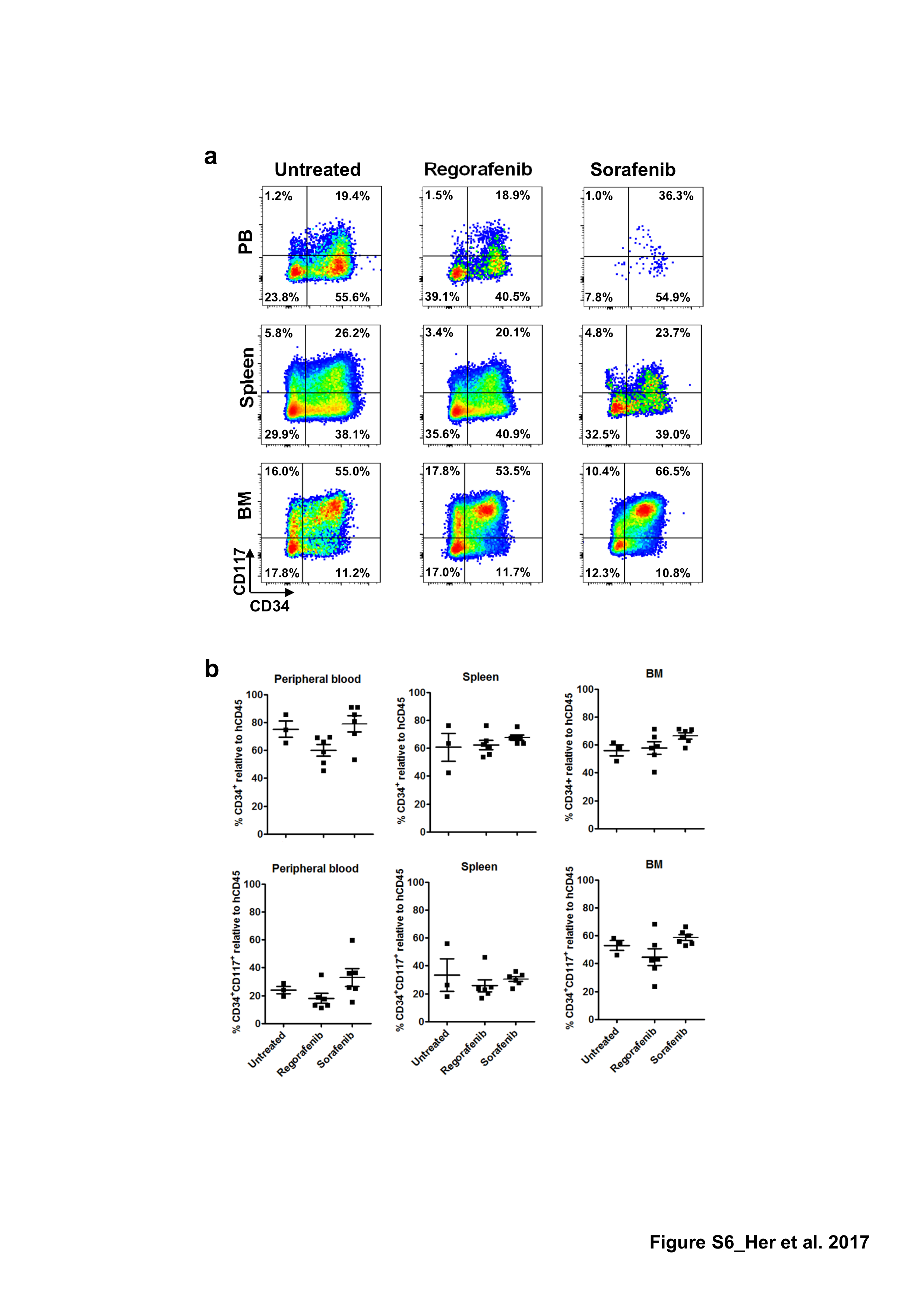

Supplement: Supplementary file 6 — Sorafenib and Regorafenib treatment has no impact on the frequency of CD34+ and CD34+CD117+ AML cells in mice. Magnetically sorted CD34+ pooled BM cells and splenocytes from secondary-engrafted NSG mice were injected intrahepatically in newborn NSG pups after sublethal irradiation (1 × 10^5^ cells per pup). Successfully engrafted mice with more than 30 human CD45+ cells per microliter of blood (between week 12 to 16 post-engraftment) were randomly assigned to either untreated (n = 3), Regorafenib (n = 6; 5 mg/kg body weight; gavage-fed once daily), or Sorafenib (n = 6; 10 mg/kg body weight; gavage-fed once daily) treatment groups and monitored for 1 month. a Representative flow cytometry plots illustrating the expression of CD34 and CD117 in human CD45 cells. b Comparison of the frequencies of total CD34+ (B, above) and CD34+CD117+ (B, below) cells relative to human CD45 cells in peripheral blood, spleen, and BM of different treatment groups after 4 weeks post-drug treatment. Data are presented as mean frequencies ± SEM. (TIFF 1403 kb) [file 13045_2017_532_MOESM6_ESM.tiff]
